# Extracellular and intraneuronal HMW-AbetaOs represent a molecular basis of memory loss in Alzheimer's disease model mouse

**DOI:** 10.1186/1750-1326-6-20

**Published:** 2011-03-06

**Authors:** Ayumi Takamura, Yasuhide Okamoto, Takeshi Kawarabayashi, Tatsuki Yokoseki, Masao Shibata, Akihiko Mouri, Toshitaka Nabeshima, Hui Sun, Koji Abe, Tsuneo Urisu, Naoki Yamamoto, Mikio Shoji, Katsuhiko Yanagisawa, Makoto Michikawa, Etsuro Matsubara

**Affiliations:** 1 Department of Alzheimer's Disease Research, Research Institute, National Center for Geriatrics and Gerontology, Aichi, Japan; 2 Department of Neurology, Institute of Brain Science, Hirosaki University Graduate School of Medicine, Aomori, Japan; 3 Immunas Pharma Incorporation, Kanagawa, Japan; 4 Department of Chemical Pharmacology, Graduate School of Pharmaceutical Sciences, Meijo University, Aichi, Japan; 5 Department of Neurology, Okayama University School of Medicine, Okayama, Japan; 6 Department of Life and Coordination-Complex Molecular Science, Institute for Molecular Science, Aichi, Japan

## Abstract

**Background:**

Several lines of evidence indicate that memory loss represents a synaptic failure caused by soluble amyloid β (Aβ) oligomers. However, the pathological relevance of Aβ oligomers (AβOs) as the trigger of synaptic or neuronal degeneration, and the possible mechanism underlying the neurotoxic action of endogenous AβOs remain to be determined.

**Results:**

To specifically target toxic AβOs *in vivo*, monoclonal antibodies (1A9 and 2C3) specific to them were generated using a novel design method. 1A9 and 2C3 specifically recognize soluble AβOs larger than 35-mers and pentamers on Blue native polyacrylamide gel electrophoresis, respectively. Biophysical and structural analysis by atomic force microscopy (AFM) revealed that neurotoxic 1A9 and 2C3 oligomeric conformers displayed non-fibrilar, relatively spherical structure. Of note, such AβOs were taken up by neuroblastoma (SH-SY5Y) cell, resulted in neuronal death. In humans, immunohistochemical analysis employing 1A9 or 2C3 revealed that 1A9 and 2C3 stain intraneuronal granules accumulated in the perikaryon of pyramidal neurons and some diffuse plaques. Fluoro Jade-B binding assay also revealed 1A9- or 2C3-stained neurons, indicating their impending degeneration. In a long-term low-dose prophylactic trial using active 1A9 or 2C3 antibody, we found that passive immunization protected a mouse model of Alzheimer's disease (AD) from memory deficits, synaptic degeneration, promotion of intraneuronal AβOs, and neuronal degeneration. Because the primary antitoxic action of 1A9 and 2C3 occurs outside neurons, our results suggest that extracellular AβOs initiate the AD toxic process and intraneuronal AβOs may worsen neuronal degeneration and memory loss.

**Conclusion:**

Now, we have evidence that HMW-AβOs are among the earliest manifestation of the AD toxic process in mice and humans. We are certain that our studies move us closer to our goal of finding a therapeutic target and/or confirming the relevance of our therapeutic strategy.

## Background

Alzheimer's disease (AD) represents the so-called "storage disorder" of amyloid β (Aβ). The AD brain contains soluble and insoluble Aβ, both of which have been hypothesized to underlie the development of cognitive deficits or dementia [1-3]. The steady-state level of Aβ is controlled by the generation of Aβ from its precursor, the degradation of Aβ within the brain, and transport of Aβ out of the brain. The imbalance among three metabolic pathways results in excessive accumulation and deposition of Aβ in the brain, which may trigger a complex downstream cascade (e.g., primary amyloid plaque formation or secondary tauopathy and neurodegeneration) leading to memory loss or dementia in AD. Accumulated lines of evidence indicate that such a memory loss represents a synaptic failure caused directly by soluble Aβ oligomers (AβOs) [4-6], whereas amyloid fibrils may cause neuronal injury indirectly via microglial activation [7]. Thus, the classical amyloid cascade hypothesis [8] underwent a modification in which the emphasis is switched to the intermediate form of Aβ such as AβOs [9-12], rather than fibrillar Aβ [7]. If this were the case, therapeutic intervention targeting AβOs may be effective in blocking this pathogenic cascade. The outcome of a recent human AN-1791 trial confirmed that plaque removal did not prevent the progression of neuronal degeneration [13], supporting this hypothesis.

However, the distinct assembly states of AβOs remain to be elucidated. Several forms of AβOs have been found to be neurotoxic, from LMW-oligomers (dimers, trimers, and tetramers) disrupting memory function [14,15], synaptic function [15,16] and long-term potentiation (LTP) [14,17], to dodecamers affecting memory [18]. In addition, Aβ-derived diffusible ligands (or ADDLs) [9,19], globulomers [11], fibrillar Aβ oligomers [20,21], and toxic soluble Aβ assembly (TAβ) [22] have been shown to be highly synaptotoxic or neurotoxic. Recently, a particular form of AβO, named the native amylospheroids [23], has been isolated from AD brains and found to induce neuronal loss through its binding to synaptic targets [24].

In this study, we chose a prophylactic passive immunization as a tool to define not only the pathological relevance of AβOs as the trigger of synaptic or neuronal degeneration, but also the possible mechanism underlying the neurotoxic action of endogenous AβOs. To address this issue, we successfully generated monoclonal 1A9 and 2C3 antibodies using a novel design method. When extracellular high-molecular-weight (HMW)-AβOs were controlled by 1A9 or 2C3 in Swedish-type amyloid precursor protein (APP) transgenic mice (Tg2576), we demonstrated that synaptic/neuronal degeneration or accumulation of intraneuronal AβOs was effectively prevented. These results argue for a role of both extracellular and intracellular HMW-AβOs in the induction and progression of synaptic or neuronal degeneration and provide a potential explanation for the extracellular one as the primary molecular basis for a toxic process.

## Results

### Generation of Aβ oligomer-specific monoclonal antibodies

**Figure 1:**
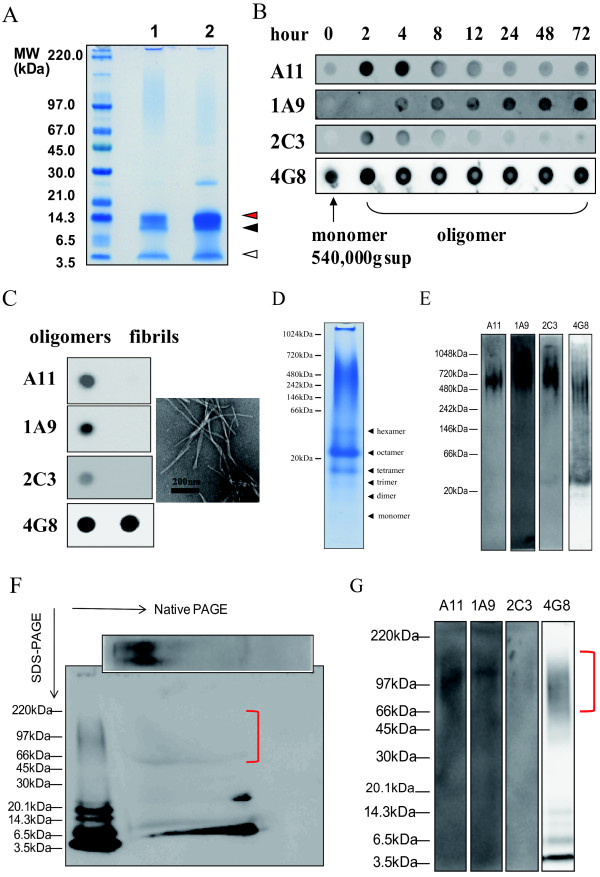
**Generation and characterization of oligomer-specific antibody**. (A) Electrophoresis of immunogen. SDS-PAGE was performed to isolate the Aβ1-42 tetramer (red closed arrowhead) alone without any contamination by the Aβ1-42 trimer (black closed arrowhead) and Aβ1-42 monomer (opened arrowhead). Lane 1, Aβ1-42 dissolved in 10 mM phosphate buffer; lane 2, Aβ1-42 dissolved in distilled deionized water. (B) Aβ1-42 oligomer formation was observed as a function of time. Aβ1-42 monomer (25 μM) incubated at 37°C for the indicated time (0 - 72 h) were spotted on nitrocellulose membrane and subjected to a dot blot assay using A11 (1:100), 1A9 (1:50), 2C3 (1:50), or 4G8 (1:1000). (C) In this dot blot assay (left half of panel C), 1 μg of soluble Aβ42 oligomers (100,000 g sup for 4-h-incubation at 37°C) and Aβ42 fibrils (100,000 g pellets for 120-h-incubation at RT) were applied on a nitrocellulose membrane and probed with A11, 1A9, 2C3, or 4G8. EM image of fibrils (right half of panel C). (D) Characterization of Aβ1-42 oligomers under nondenaturing conditions. Aβ1-42 monomer (25 μM) incubated at 37°C for 4 h was separated on 16% BN-PAGE. (E) Separated peptides under nondenaturing conditions were also subjected to immunoblot analysis using A11, 1A9, 2C3, and 4G8. (F) The 100000 g sup of 4-h-incubated mixture of Aβ1-42 monomer (25 μM) was subjected to two-dimensional native/SDS-PAGE, followed by 4G8-immunoblot analysis. SDS-stable 15~40-mers are indicated (] (red)). (G) Immunodetection of 4-h-incubated mixture of Aβ1-42 monomer (25 μM) under denaturing conditions probed with A11, 1A9, 2C3, and 4G8. SDS-stable 15~40-mers are indicated (] red).

Since the removal of AβMs is critical for the preparation of antigens to obtain AβO-specific antibodies, we isolated SDS-stable Aβ tetramers alone without any contamination of Aβ trimers and AβMs by SDS-PAGE (Figure 1A). After *in vivo *immunization with the gel containing the Aβ tetramer alone, positive hybridoma supernatants were screened by dot blot analysis. Among positive supernatants (16/400, positive % = 4%), two clones, namely, 1A9 and 2C3, were generated from a mouse that produced IgG2b. As shown by dot blot analysis, both 1A9 and 2C3 recognized soluble AβOs (100,000 g supernatant (sup) of 4-h-incubated mixture, Figure 1B and 1C), not AβMs (560,000 g sup of seed-free preparation, Figure 1B) or Aβ fibrils (100,000 g pellet of 120-h-incubated mixture, Figure 1C), in contrast with 4G8 (Figure 1B and 1C). The generation of A11- or 2C3-immunoreactive oligomers preceded that of 1A9-immunoreactive oligomers (Figure 1B). The 1A9 conformer displayed a different pattern of its time course, showing the highest stability in the oligomeric assembly state (Figure 1B).

We further assessed the precise size of 1A9- or 2C3-immunoreactive AβOs. Blue native polyacrylamide gel electrophoresis (BN-PAGE), a charge shift method of electrophoresis, was carried out to determine the molecular mass and native oligomeric status of the 4-h-incubated mixture. As shown in Figure 1D, the native Aβ species exhibit Aβ monomers, low-molecular-weight (LMW)-oligomers (dimers, trimers, tetramers, pentamers, and heptamers), and a high-molecular-weight (HMW)-oligomeric smear with molecular masses ranging in sizes from 66 to 720 kDa (Figure 1D). Immunoblot analysis employing monoclonal 1A9 revealed its immunoreactivity with Aβ species corresponding to HMW-oligomeric smear of 480 to 1048 kDa (Figure 1E), whereas 2C3 immunoreacted with Aβ species corresponding to a broad smear of 146 to 1048 kDa plus minute amounts of pentamers (Figure 1E). The anti-oligo A11 immunoreacted with native Aβ species corresponding to a HMW-smear ranging in sizes from 242 to 1000 kDa (Figure 1E), whereas monoclonal 4G8 detected Aβ oligomers larger than tetramers (Figure 1E). The combination of two-dimensional native/SDS-PAGE and 4G8-immunoblot analysis revealed that 2% SDS disaggregates HMW-AβOs down to monomers and LMW-oligomers in addition to SDS-stable HMW-oligomers with molecular masses corresponding to those of 8~40-mers (Figure 1F). These findings suggest that the assignment of oligomer size is dependent on the method of evaluation. Indeed, monoclonal 4G8 detected all the Aβ species separated, whereas anti-oligo A11, 1A9, or 2C3 immunoreacted with SDS-stable 15-mers to 40-mers under our denaturing conditions tested (Figure 1G), indicating that 1A9 or 2C3 is indeed specific to AβOs.

### Characterization of neurotoxic AβOs

**Figure 2:**
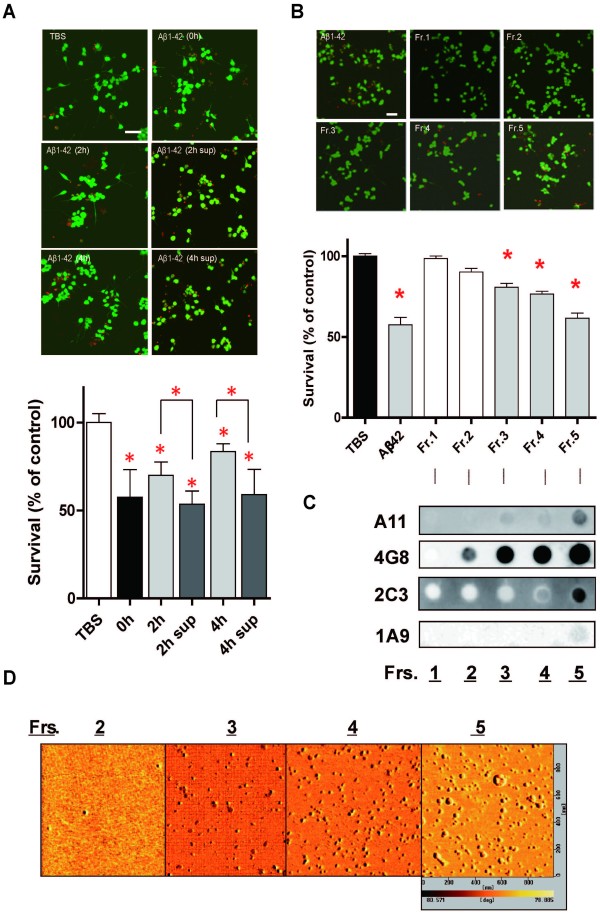
**Biophysical and structural characterization of neurotoxic Aβ assembly**. (Upper half of panel A) Representative calcein AM/PI stainings of NGF-treated PC12 (PC12N) cells treated at 37°C for 48 h with: TBS alone; 0-h preincubated Aβ1-42 (0 h); 2-h preincubated Aβ1-42 (2 h); 540,000 *g *supernatant obtained from 2 h (2 h sup); 4-h preincubated Aβ1-42 (4 h); 540,000 *g *supernatant obtained from 2 h (4 h sup). Green staining for viable cells versus red staining for dead cells. Resultant cell viability for each treatment is shown in lower half of panel A. Experimental results were analyzed by one-way ANOVA, followed by Tukey's test for posthoc analysis: statistical significance compared with TBS alone (*p <0.0001). Scale bar = 50 μm. (B) The seed-free Aβ1-42 (25 μM) was subjected to a series of membrane ultrafiltration steps with molecular cutoffs at 3, 10, 30, and 100 kDa. The resultant four filtrates and one retentate were designated as Fr. 1 (<3 kDa), Fr. 2 (3-10 kDa), Fr. 3 (10-30 kDa), and Fr. 4 (30-100 kDa), and final retentate Fr. 5 (>100 kDa). The upper half of panel B shows the representative calcein AM/PI stainings of NGF-treated PC12 (PC12N) cells incubated at 37°C for 48 h with seed-free unfractionated Aβ1-42 or five fractions (25 μM each). Resultant cell viability for each treatment is shown in the lower half of panel B. Experimental results were analyzed by one-way ANOVA, followed by Dannett's test for posthoc analysis: statistical significance compared with TBS alone (*p <0.001). (C) Dot blot analysis of five fractions (Frs. 1-5). The blots were reacted with A11, 1A9, 2C3, and 4G8. (D) Amplitude AFM images (2 μm × 2 μm) of four fractions (Frs. 2-5). All AFM images were taken on a mica surface.

We further assessed the morphology of neurotoxic AβOs. After 0-h-, 2-h- or 4-h-incubation at 37°C, we tested the bioactivity of each incubated mixture (25 μM) by incubating NGF-differentiated PC12 (PC12N) cells [22] at 37°C for 48 h. Surprisingly, Live/Dead two-color fluorescence assay [22] revealed that 2-h- or 4-h-preformed Aβ42 assembly decreased the toxic activity, whereas depletion of "insoluble" AβOs and concentration of "soluble" AβOs fully restored the toxic activity, similarly to that of Aβ42 assembly formed from seed-free fresh peptide (0 h) (Figure 2A). These data indicated that seed-free fresh peptide (0 h) is the best source of *de novo *formation of neurotoxic AβOs. We isolated soluble Aβ species originated from seed-free fresh peptide by ultrafiltration and molecular sieving, which allowed us to separate the toxic Aβ42 peptide into five fractions (Figure 2B). To verify the size distribution of toxic Aβ42, PC12N cells were exposed to each fraction (25 μM) at 37°C for 48 h (Figure 2B). The level of LDH released from PC12N cells, when treated with each fraction, was similar to that treated with TBS alone. Live/Dead two-color fluorescence assay [22] revealed the toxicity of unfractionated Aβ42 or Frs. 3-5 (Figure 2B, F (6/77) = 39.85, *p *< 0.0001), suggesting that toxic oligomers are at least larges trimers. Neurotoxic AβOs were then subjected to further dot blot immunoanalysis without SDS to determine their size, in which their "native" conformations are supposed to be maintained (Figure 2C). Monoclonal 4G8 detected Aβ species in Frs. 2-5. The anti-oligomer A11 unequivocally reacted with toxic oligomeric conformers in Frs. 3-5. In contrast, 2C3 immunoreactivities were observed in Frs. 4 and 5, whereas minute amounts of 1A9 conformers were detected in toxic Fr. 5 alone. Taken together, these data suggest that both 1A9 and 2C3 toxic conformers are larger than 100 kDa and 30 kDa, respectively. Atomic force microscopy (AFM) [25] of toxic fractions revealed structures of different sizes and morphologies, including relatively compact spherical particles approximately 5-10 nm in diameter, large spherical particles roughly 25-50 nm in diameter, and an annular pore like structure 25 nm in inner and 75 nm in outer diameters (Figure 2D).

### 1A9 and 2C3 immunoreactivitiy in human tissue

**Figure 3:**
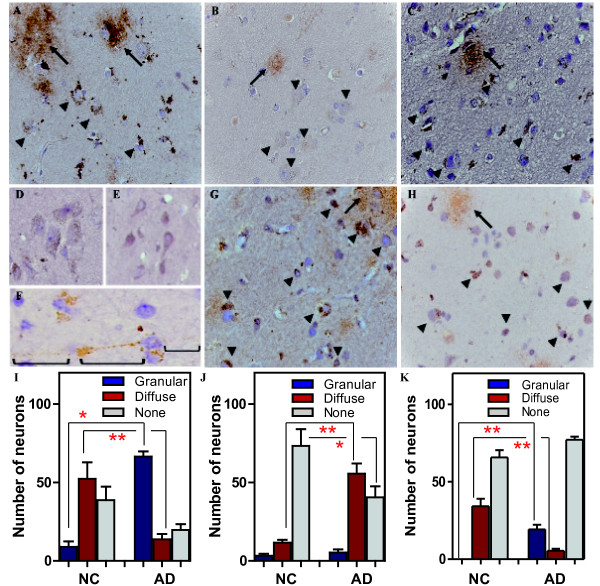
**Immunolabelling characteristics of 1A9, 2C3, A11, 6E10 and 4G8 in human brains**. Typical AβO immunolabelling is observed in diffuse plaques (arrow) and the perikaryon of pyramidal neurons (arrow head) as dense granules (A and C) or diffuse staining (B) in AD brains (400X): 1A9 (1:50, panel A), 2C3 (1:50, panel B), and A11 (1:250, panel C). In control brains (D), 1A9 disclosed small granular intraneuronal staining in isolated clusters of pyramidal neurons (400X). (E) Diffuse intraneuronal staing is a characteric feature of control brains (A11, 1:250; 400X). (F) 1A9-positive granules were often observed in dendrires (⊔) in AD brains (400X). (G-H) 4G8 (1:100) and 6E10 (1:100) immunoreactivities were also observed in neurons (arrow head) and diffuse plaques (arrow) of AD brains, respectively (400X). (I-K) Determinations of number of intraneuronal AβO immunolabelling in human brains: 1A9 (panel I), 2C3 (panel J), and A11 (panel K). Designations used herein were as follows: Granular, dense granule staining; Diffuse, diffuse staining; None, no staining; NC, normal control; AD, Alzheimer's disease. Experimental results of staining pattern were analyzed by one-way ANOVA, followed by Dannett's test for posthoc analysis: statistical significance compared with NC (*p < 0.01, **p < 0.001).

To investigate the distribution of 1A9 and 2C3 staining in human tissue, immunohistochemistry was performed on brain tissues obtained from 4 AD and 3 age-matched normal cases. The use of monoclonal 1A9 (Figures 3A and 3I) and, to a lesser extent, monoclonal 2C3 (Figures 3B and 3J) and polyclonal A11 (Figures 3C and 3K) revealed that AβOs are highly localized within pyramidal neurons. Within these cells, AβOs accumulated as densely packed granules in the perikarya (Figures 3A-C). Some of the diffuse plaques were stained by 1A9, but diffuse deposits were poorly stained by 2C3 and A11 (Figures 3A-C). The majority of 1A9-stained pyramidal neurons exhibited atypical, eccentric large nuclei with abnormal chromatin morphology and distributions, features indicative of impending neuronal degeneration (Figures 3A and 3I). Such abnormalities were less evident in 2C3- or A11-stained pyramidal neurons (Figures 3B, C, J and 3K). Although age-matched control brains sometimes contained isolated clusters of AβO-burned neurons (Figures 3D), the number of neurons involved and the amount of AβOs accumulated within these cells were much lower than those in AD brains (Figures 3D and 3I). Typically, 1A9-immunoreactive granules in control brains were relatively small, numerous, and rather uniformly distributed throughout the perikaryon of pyramidal neurons and the proximal portion of apical dendrites (Figure 3D). In addition, intraneuronal AβOs were diffusely distributed throughout the perikaryon of pyramidal neurons, which appears to be a characteristic feature in control brains (Figures 3E and 3I-K). In AD brains, intraneuronal AβOs were sequestered into large, densely packed aggregates in the dendritic trunk and/or branches, and axons (Figures 3A-C). Interestingly, some showed multiple dot-like accumulation of 1A9-immunoreactive AβOs arranged in tandem along apical dendritic shafts, which were focally swollen with an accumulation of 1A9-immunoreactive AβOs (Figure 3F). The presence of intraneuronal Aβ was further supported by the staining by the widely used 4G8 (Figure 3G) and 6E10 (Figure 3H).

### Extracellular AβOs are uptaken by neurons

**Figure 4:**
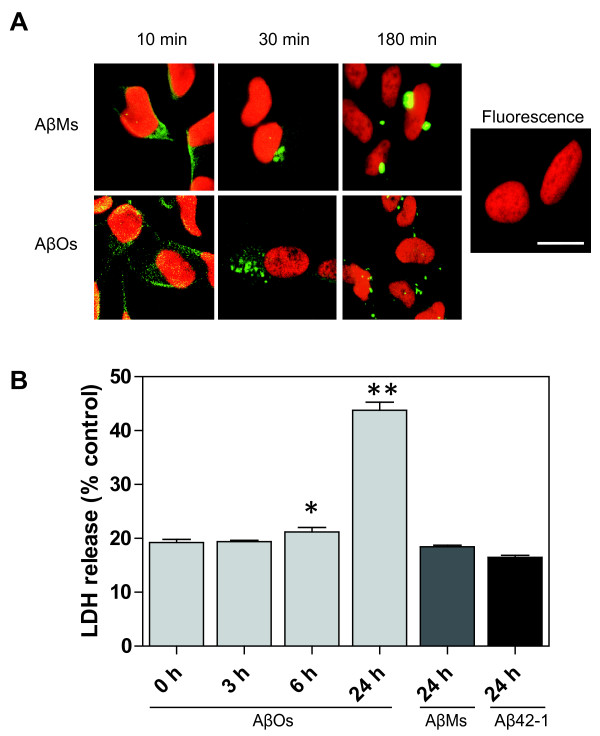
**Cell uptake of neurotoxic AβOs**. (A) SH-SY5Y cells were exposed to Fluor™488 alone, 5 μM HiLyte Fluor™488-labeled AβMs or AβOs (green) at 37°C for 10, 30, and 180 min. AβMs: 10 kDa-filtrate; AβOs:30 kDa-retentate. Nuclear staining (7-AAD) is shown in red. Vesicular uptake was observed with AβOs, but not with AβMs and Fluor™488 alone. (B) The level of LDH released from SH-SY5Y cells treated for the indicated times (0, 3, 6, and 24 h) with 5 μM AβOs. In the case of 5 μM synthetic Aβ42-1 and AβMs, LDH assay was done for 24 h. Each value indicates the percentage level of LDH released following treatment with incubation mixtures relative to the level of LDH released following treatment with Triton X-100. Each column indicates average ± S.D. The *p *value was determined by one-way ANOVA, followed by Dannett's test for posthoc analysis: statistical significance compared with AβMs alone (*p < 0.05, **p < 0.001).

To gain further insight into the link between extracellular and intraneuronal AβOs, neuroblastoma (SH-SY5Y) cells were incubated with 5μM HiLyte Fluor™488-labeled AβMs, AβOs, or Fluor™488 alone. Immunocytochemical analysis showed that fluorescence-labeled AβOs, not AβMs, bind to neuronal membrane for 10 min, followed by accumulation of intraneuronal AβOs for 0.5 or 3 hr (Figure 4A). Vesicular uptake was not observed with fluorescence alone (Figure 4A). Only when Fluor™488-labeled AβOs were added to SH-SY5Y cells, the LDH release assay [22] of cultures of SH-SY5Y cells revealed that the level of released LDH increased as a function of time (Figure 4B), suggesting that accumulation of intraneuronal AβOs induces neuronal cell death. The cell death was not detectable in the case of synthetic Aβ 42-1 and AβMs (Figure 4B).

### Monoclonal 1A9 or 2C3 immunotherapy protects Tg2576 from memory impairment

**Figure 5:**
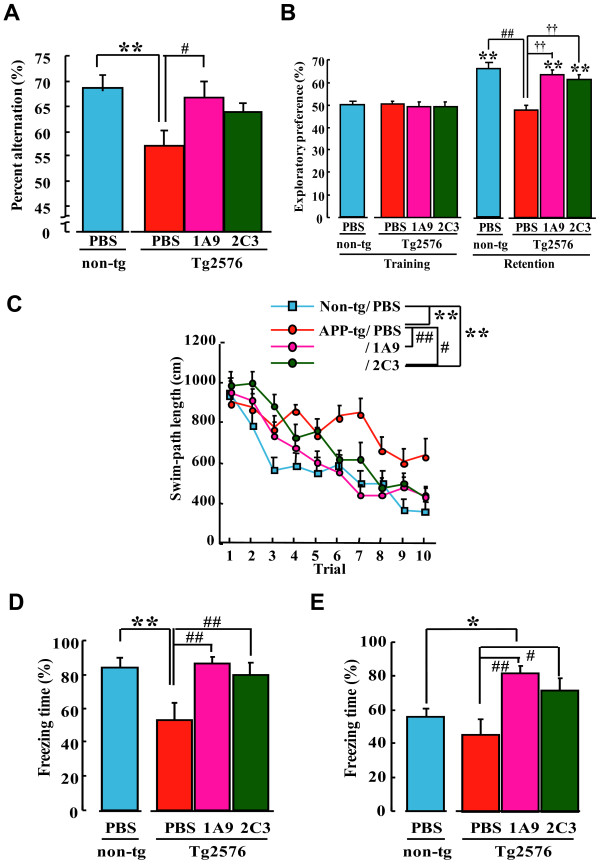
**Passive immunization protects Tg2576 mice from memory deficits**. Tg2576 mice at 13 months of age in three groups were studied: PBS-treated non-Tg mice, n = 14; PBS-treated Tg2576 mice, *n *= 10; 1A9-treated mice, *n *= 13; 2C3-treated mice, *n *= 12. Values indicate the mean ± SEM. (A) Y-maze test. Spontaneous alternation behavior during an 8-min session in the Y-maze task was measured in each group. Results of one-way ANOVA were as follows: F(3, 45) = 2.99, p < 0.05, **p < 0.05 vs PBS-treated non-Tg mice, #p < 0.05 vs PBS-treated Tg2576 mice. (B) Novel object recognition test. The retention session was carried out 24 h after the training. Exploratory preference during a 10-min session in the novel-object recognition test was measured in each group. Results of the two-way ANOVA were as follows: training/retention, F(1, 90) = 58.19, p < 0.01; animal group, F(3, 90) = 6.18, p < 0.01; interation of training/retention with animal group, F(3, 90) = 7.57, p < 0.01; **p < 0.01 vs corresponding trained mice. ##p < 0.01 vs PBS-treated non-Tg mice, **p < 0.01 vs PBS-treated Tg2576 mice. (C) Swimming-path length during a 60-s session in the water maze test was measured in each group. Results of the two-way ANOVA were as follows: trial, F(9, 450) = 25.51, p < 0.01; animal group, F(3, 450) = 14.85, p < 0.01; interaction of trial with animal group, F(27, 450) = 1.36, p = 0.11; **p < 0.01 vs PBS-treated non-Tg mice, #p < 0.05, ##p < 0.01 vs PBS-treated Tg2576 mice. Conditioned fear learning test: context-dependent (D) and cue-dependent (E) freezing times were measured. The results of one-way ANOVA were as follows: context-dependent test, F(3, 45) = 6.19, p < 0.01; cue-dependent test, F(3, 45) = 5.41, *p < 0.05; **p < 0.01 vs PBS-treated non-Tg mice, #*p *< 0.05, ##*p *< 0.01 vs PBS-treated Tg2576 mice.

Using active 1A9 and 2C3 antibodies, we next evaluated whether a specific control of endogenous AβOs *in vivo *would be sufficient to prevent the disruption of neuronal function leading to memory loss. To assess this possibility, a long-term low-dose prophylactic study using 1A9 and 2C3 was designed instead of a therapeutic approach that was used previously by other scientists [26,27]. Tg2576 mice were injected with 2C3 (n = 12), 1A9 (n = 13), or PBS (n = 10) into the tail vein (0.4 mg/kg/week) from 4 months of age (that is, about 2 months before the onset of memory loss) until 13 months of age (when memory loss and amyloid plaque formation are already well established). Memory functions were measured in four behavioral paradigms, as described previously [28]: (1) short-term memory in the Y-maze test (Figure 5A); (2) object recognition memory in a novel object recognition test (Figure 5B); (3) spatial memory in the water maze test (Figures 5C); and (4) associated emotional memory in the contextual fear learning test (Figures 5D and 5E). Untreated Tg2576 mice (n = 14) showed a significantly poorer behavioral performance than untreated wild-type mice in all the behavioral paradigms tested (Figure 5A-5E). Tg2576 mice treated with 1A9 and, to a lesser extent, 2C3 showed significantly better behavioral performance than untreated Tg2576 mice in all the behavioral paradigms tested (Figures 5A-5E). Unlike that of untreated Tg2576 mice, the performance of 1A9- or 2C3-treated Tg2576 mice was indistinguishable from that of untreated wild-type mice (n = 14) that had been previously tested in these tasks at this age [28], indicating that both short-term and long-term memories were well preserved in 1A9- or 2C3-treated Tg2576 mice.

### Monoclonal 1A9 or 2C3 immunotherapy protects Tg2576 from synaptic degeneration and neuronal degeneration

**Figure 6:**
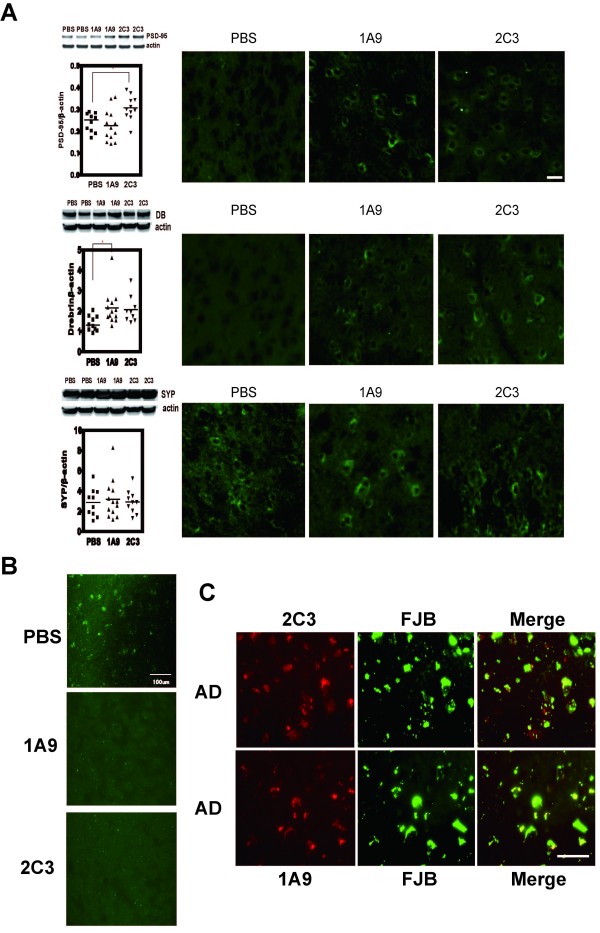
**Passive immunization protects Tg2576 mice from synaptic or neuronal degeneration**. (**A**) Saline-insoluble, SDS-extractable synaptic proteins were examined by Western blot analysis and probed for PSD95 (1:250), drebrin (DB, 1:100), and synaptophysin (SYP, 1:2000). The *p *value was determined by one-way ANOVA: *p < 0.05. Representive Confocal immunofluorescence microscopy images of Tg2576 mouse brain. Sections of the cortex of the untreated (PBS)-, 1A9-treated, or 2C3-treatedTg2576 mouse brain were immunostained with PSD-95 (Upper part of panel A), Derbrin (DB, Middle part of panel A), synaptophysin (SYP, Lower part of panel A). Scale bar = 30 μm. (**B**) Confocal immunofluorescence microscopy images of Tg2576 mouse brain. Sections of the hippocampus of the untreated (PBS)-, 1A9-treated, or 2C3-treatedTg2576 mouse brain were immunostained with Fluoro Jade B (1:50000). Scale bar = 100 μm. (**C**) Representive Confocal immunofluorescence microscopy images of AD brain. Sections of the hippocampus of AD brains were immunostained with Fluoro Jade B. Scale bar = 20 μm.

The synaptoprotective effect was also confirmed at the postsynapse level, not at the presynapse level, at which the relative intensities of PSD-95 (Figure 6A) to actin were significantly higher in 2C3-treated Tg2576 mice than in untreated Tg2576 mice. This is not the case with synaptophysin (Figure 6A). Thus, 2C3 indeed protect Tg2576 mice from postsynaptic degeneration. In addition, the intensities of drebrin (Figure 6A) relative to those of actin were significantly higher in 1A9-treated Tg2576 mice, indicating that 1A9 protects Tg2576 mice from the degeneration of dendritic spines. This assumption was further supported by image analysis of synaptophysin, PSD-95, or drebrin after immunofluorescence microscopy analysis (Figure 6A).

To further assess the neuroprotective effect of 1A9 or 2C3 immunotherapy, Fluoro-Jade B (FJB) binding assay, which specifically detects degenerative neurons, [29], was performed. As depicted in Figure 6B, abundant FJB-positive neurodegenerative neurons were evident in untreated Tg2576 mouse brains. In contrast, such neurodegenerative neurons were negligible in 1A9- (Figure 6B) or 2C3-treated Tg2576 mouse brains (Figure 6B), indicating that 1A9 or 2C3 immunotherapy protects Tg2576 mice from neuronal degeneration.

Double-labeling analysis of AD brains (Figure 6C) revealed that 1A9- or 2C3-positive neurons were degenerated as proven by Fluoro-Jade B(FJB) binding, indicating that the accumulation of intraneuronal AβOs is closely associated with neuronal degeneration (Figure 6C).

### Monoclonal 1A9 or 2C3 immunotherapy protects Tg2576 from the accumulation of AβOs

**Figure 7:**
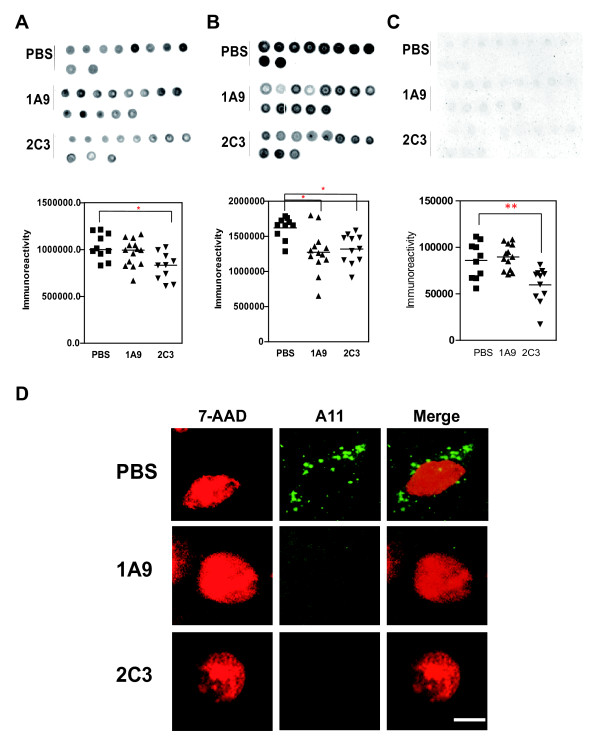
**Passive immunization protects Tg2576 mice from accumulation of intraneuronal AβOs**. Dot blot immunoassay for A11-immunoreactive AβOs in saline-soluble extracts (**A**), saline-insoluble, SDS-extractable extracts (**B**), and SDS-insoluble, FA-extractable extracts (**C**) obtained from untreated, 1A9-treated, or 2C3-treated mice (30 μg of total extracted brain protein per dot). Results from densitometric imaging of these same samples. The line indicates the mean of each set. The *p *value was determined by one-way ANOVA: *p < 0.05, **p < 0.001. (**C**) Confocal immunofluorescence microscopy images of Tg2576 mouse brain. Sections of the hippocampus of the Tg2576 mouse brain were doubly immunostained with 7-AAD (red, 1:50) and anti-A11 antibody (green, 1:250). Scale bar = 10 μm.

To gain further insight into the neurotoxic action of AβOs, we determined whether 1A9 and 2C3 immunotherapies targeting endogenous AβOs can prevent the accumulation of extracellular or intracellular AβOs *in vivo*. A11-dot immunoblot analysis revealed that 2C3 immunization had a significant preventive effect (*p *< 0.05 versus untreated group, post-hoc test) on the level of saline-soluble AβOs (Figure 7A) which represents extracellular soluble AβOs. In contrast, 2C3 and 1A9 immunizations resulted in a significant reduction in the level of SDS-extractable AβOs (Figure 7B), which represents mainly intracellular AβOs, owing to the specificity of 1A9 or 2C3 towards intracellular Aβ assembly (see Figure 3). In the case of SDS-insoluble, FA-extractable fractions which represent highly insoluble AβOs, A11-dot immunoblot analysis showed that 2C3 immunization had a significant preventive effect (*p* <0.001 versus untreated group, post-hoc test) (Figure 7C). In accordance with these data, double-labeling analysis revealed that 1A9 and 2C3 immunotherapies prevent the accumulation of A11-immunoreactive granules in the neurons regardless of the abundant A11-specific fluorescence found in untreated mice (Figure 7D).

## Discussion

The development of AβOs-selective antibodies has greatly facilitated the understanding of *in vivo *relevance of endogenous AβOs-mediated synaptic failure or neuronal degeneration. To prove this issue, a prophylactic study to control endogenous AβOs using AβOs-selective antibodies is required. Several conformation-dependent antibodies such as oligomer- or fibril-specific antibodies were reported previously [12,20,21,27,30,31], but none of them was examined for this purpose. In the current study, we successfully generated monoclonal oligomer-specific 1A9 and 2C3 using a novel design concept. Monoclonal 1A9 recognizes HMW-oligomers (100~230-mers), whereas 2C3 recognizes LMW- and HMW-oligomers ranging in sizes larger than pentamers (5~230-mers). In support of a previous report [12], prefibrillar oligomer-selective A11 appears to be specific to HMW-oligomers. Under conditions of SDS-PAGE, 1A9-, 2C3-, or A11-oligomeric conformers were consistently detected at 70-180 kDa corresponding to 15~40-mers. Note that neither 1A9 nor 2C3 reacted with monomers and fibrils. In spite of heterogeneity in size, AFM clearly demonstrated that the toxic 1A9-, 2C3-, or A11-oligomeric conformers display relatively compact spherical particles, not fibrilar structure.

Using these oligomer-selective 1A9 and 2C3, we found that the majority of AβOs exclusively accumulated in neurons, whereas the degree of staining of diffuse plaques varied among antibodies tested (1A9>2C3 = A11). Under our conditions tested, A11-immunoreactivity is occasionally found in small diffuse plaques, in contrast with the finding of a previous report [12]. These findings are direct evidence that heterogeneous oligomeric conformers exist as a distinct entity in both extracellular and intraneuronal deposits in human brains. Furthermore, the change of nuclear appearance in AβOs-burned neurons is highly indicative of impending neuronal degeneration: 1A9-positive AβOs may be associated with the most severe neuronal degeneration among the three anti-oligomer-specific antibodies tested. FJB binding assay, which specifically detects degernerating neurons [29], also confirmed that 1A9- or 2C3-burned neurons in AD brains were unequivocally FJB-positive, indicating that intraneuronal accumulation of AβOs is closely associated with neuronal degeneration.

Using above-mentioned 1A9 and 2C3, we evaluated whether a specific control of endogenous, extracellular AβOs *in vivo *would be sufficient to prevent the disruption of neuronal function leading to memory loss. Our *in vivo *investigation demonstrated that immunized subjects have less intraneuronal accumulation of AβOs and fewer degenerating neurons than untreated controls. In addition, 2C3 and 1A9 protected Tg2576 mice from postsynaptic degeneration and from the degeneration of dendritic spines, respectively. These results place both extracellular [22,32] and intraneuronal AβOs [33,34] centrally within the mechanisms mediating AβO-induced neuronal dysfunction leading to memory loss [26,27]. In support of these data, our in vivo investigations clearly demonstrated that 1A9- and 2C3-treated Tg2576 mice aged 13 months showed cognitive performance superior to that of untreated Tg2576 mice, and, ultimately, performed better than and/or as well as untreated wild-type mice. It is unlikely that the impaired performance of Tg2576 mice in learning and memory tests is due to changes in motivation or sensory motor function, because the purpose of each behavioral test is different, and different skills are required for a good performance in each test. There were no differences in locomotor activity and the total time spent exploring objects in the novel object test between the wild-type and Tg2576 mice. Thus, it is likely that endogenous oligomeric 1A9- or 2C3-conformer is not only generated in Tg2576 mice, but is also actually a bioactive molecule *in vivo*, and its selective immunoneutralization by systemic administration of 1A9 or 2C3 is sufficient to prevent either short-term or long-term memory loss. This *in vivo *neuroprotective activity of 1A9 appeared to be superior to that of 2C3, supporting our current finding that neuronal degeneration in AD brains is more severe in 1A9-burned neurons than in 2C3-burned neurons. Recently, the generation of a new mouse model expressing only AβOs in neurons has demonstrated that endogenous AβOs are neurotoxic *in vivo *inducing synaptic alteration, abnormal tau phosphorylation, glial activation, and neuronal loss [35], supporting the pathological relevance of AβOs as shown herein. Taken together, the results from this study indicate that both extracellular and intraneuronal AβOs represent a molecular basis of memory loss *in vivo*. Additional studies that attempt to identify the cellular and molecular targets on the cellular surface with which 1A9 or 2C3 interacts may yield insights into the mechanisms underlying the synaptotoxic or neurotoxic effects of AβOs or synaptoprotective or neuroprotective effect of 1A9 or 2C3.

## Conclusions

We herein performed a hypothesis-driven, proof of concept study to prove the relevance of the *in vivo *Aβ oligomer hypothesis using monoclonal antibodies specific to AβOs generated using a novel design method. We found that AβOs are not only the real memory-relevant molecules, but also the real culprits of neuronal degeneration. Now, we have evidence that AβOs are among the earliest manifestation of the AD toxic process in mice and humans. We are certain that our studies move us closer to our goal of finding a therapeutic target and/or confirming the relevance of our therapeutic strategy.

## Methods

### Generation of monoclonal 1A9 and 2C3

Synthetic Aβ1-42 (r-peptide, Osaka, Japan) was dissolved in distilled, deionized H_2_O, or 10 mM phosphate buffer at 250 μM, and allowed to incubate at 37°C for 18 h. Twenty microgram of preincubated Aβ1-42 was separated by NuPAGE 4-12% Bis-tris glycine gels, followed by CBB staining. Balb-c mice were immunized by injection with 2.5 μg of Aβ1-42 tetramer alone, which was excised from the gel and emulsified with complete Freund's adjuvant, into foot pads, followed by six additional injections. Inguinal lymphonode was used to generate hybridomas by fusion with Sp2/O-Ag14 myeloma cells with polyethylene glycol 1500. Initial screening was performed by dot blot analysis, applying 2.5 μl of seed-free fresh or 18-h preincubated Aβ1-42 (2.5 ng/dot) to a nitrocellulose membrane [22]. The blots were then allowed to dry and blocked with 5% low-fat milk and 1% BSA in PBS containing 0.05% Tween-20 (PBST) and incubated with culture medium supernatant, followed by horseradish peroxidase (HRP)-labeled goat anti-mouse or anti-rabbit F(ab')_2 _antibody (1:3000; Amersham). Dot immunoblots were visualized with an ECL kit using LAS3000 mini (Fujitsu, Tokyo, Japan).

### Preparation of Aβ1-42 peptide

Synthetic Aβ1-42 was dissolved in 0.02% ammonia solution at 250 μM. To obtain seed-free Aβ42 solutions (540,000 g sup), the prepared solutions were centrifuged at 540,000 *g *for 3 h using an Optima TL ultracentrifuge (Beckman, USA) to remove undissolved peptides, which can act as preexisting seeds. The supernatant was collected and stored in aliquots at -80°C until use. Immediately before use, the aliquots were thawed and diluted with TBS (150 mM NaCl and 10 mM Tris-HCl, pH 7.4). For time- course experiments, 540,000 g sup was incubated for an indicated time (0-72 h), and soluble AβOs were retained after 100,000 g ultracentrifugation for 1 h, followed by dot immunoblot analysis (2.5 ng/dot) [22]. Because the 4-h-incubated mixture is suitable for the characterization of soluble AβO standards, we used it in further experiment instead of the 18-h-incubated mixture used in the first screening. To further assess whether monoclonal 1A9 or 2C3 recognizes Aβ fibrils, seed-free Aβ42 solutions (25 μM) were incubated for 5 days at room temperature. Electron-microscopy-confirmed Aβ fibrils were subjected to dot immunoblot analysis (2.5 ng/dot) [22].

### Electron microscopy (EM)

For electron microscopy, samples were diluted with distilled water and spread on carbon-coated grids. The grids were negatively stained with 1% phosphotungstic acid and examined under a Hitachi H-7000 electron microscope (Tokyo, Japan) with an acceleration voltage of 77 kV.

### BN-PAGE and two-dimensional Natie/SDS-PAGE

BN-PAGE analysis was performed following the manufacture's instruction (Invitrogen, Carlsbad, CA): 4%, 6%, 8%, 10%, 12%, 14%, 16%, 18%, 20%, and 4-16% Novex^® ^Bis-Tris gel was used. The apparent molecular masses of LMW-oligomers were calculated from the Ferguson plots with known molecular mass standards (α-lactalbumin, 14.2 kDa; carbonic anhydrase, 29 kDa; chickin egg albumin, 45 kDa; bovine serum albumin, 66 kDa monomer, and 132 kDa dimer; urease, 272 kDa monomer and 545 kDa dimer) (Sigma). For two-dimensional native/SDS-PAGE, one lane was excised from the gel and applied directly for SDS-PAGE. Immunoblot analysis was performed as described previously [36].

### Aβ-induced toxicity assay

We conducted the Aβ-induced toxicity assay according to previously published methods [22]. Briefly, rat pheochromocytoma 12 (PC12) cells were cultured in DMEM (Invitrogen, Carlsbad, CA) supplemented with 10% heat-inactivated horse serum (Invitrogen) and 5% FBS (Invitrogen). For their differentiation, PC12 cells were plated on 10-cm^2 ^poly-L-lysine-coated (10 mg/ml) dishes at a density of 20,000 cells/cm^2 ^and cultured for 6 days in DMEM supplemented with 100 ng/ml nerve growth factor (NGF; Alomone Labs, Jerusalem, Israel) (PC12N). Basically, toxicity was assessed using different Aβ conformers: seed-free Aβ1-42 vs. 2-h- or 4-h-preincubated Aβ1-42 with or without 540,000 g ultracentrifugation for 1 h. PC12N was exposed to seed-free or preincubated Aβ1-42 at 25 μM for 48 h at 4°C. Toxicity was assessed by Live/Dead two-color fluorescence assay (Molecular Probes, Eugene, OR) or CytoTox 96 Non-Radioactive Cytotoxicity Assay Kit (Promega, Madison, DI) in accordance with the manufacturer's instructions as described previously [22].

### Ultrafiltration and molecular sieving (UF/MS)

To characterize the size of *de novo *formed toxic AβOs at 25 μM, serial ultrafiltration using Microcon^® ^3-, 10-, 30-, and 10-kDa cut-off membranes (Millipore Corp. Billerica, MA) was performed to prepare four filtrates (12,000 g-centrifuge for 90 min, Fr. 1, >3 kDa; 12,000 g-centrifuge for 60 min, Fr. 2, 3-10 kDa; 12,000 g-centrifuge for 30 min, Fr. 3, 10-30 kDa; 12,000 g-centrifuge for 15 min, Fr. 4, 30-100 kDa) and one retentate (Fr. 5, >100 kDa). Each fraction at 25 μM was subjected to Aβ-induced toxicity assay as described above. PC12N cells were exposed to each fraction and the toxic fraction was assessed as described above. The distribution of the A11, 1A9, 2C3, or 4G8 conformer was characterized by dot blot analysis as described above. To determine the morphology of toxic oligomers, each fraction was also subjected to atomic force microscopy (AFM).

### Atomic force microscopy (AFM)

AFM assessment was performed as reported previously [25]. Briefly, samples were dropped onto freshly cleaved mica. After allowing them to stand for 1 h, following by washing with water, the samples were assessed in a solution using a Nanoscope IIIa (Digital Instruments, Santa Barbara, CA, USA) set at tapping mode. OMCL-TR400PSA (Olympus, Japan) was used as a cantilever. Consecutive scans were monitored until distortion due to creep or shifts in the slow scan direction were negligible before collecting scans at sizes of 2 μm with the maximum 512 × 512 pixel resolution.

### Immunohistochemistry

The left hemispheres of the brains of Tg2576 mice were sagitally cut into 30-μm-thick sections using a freezing microtome (RM 2145; Leika, Wetzlar, Germany). The tissue blocks from human subjects (4 AD subjects and 3 normal controls) or mice were fixed in 4% paraformaldehyde with 0.1 M phosphate-buffered saline (pH 7.6) and embedded in paraffin wax. After deparaffinization, heat-induced antigen retrieval was achieved by boiling sections for 10 min in a microwave oven in 0.01 M citrate buffer pH6.0, followed by 3 min incubation in 99% formic acid and then blocking of endogenous peroxidase. Then sections were subsequently incubated for 1 hour with primary antibody diluted in blocking buffer with normal goat or horse serum (2%), and after washing for 1 hour, with a secondary antibody in the same buffer. All incubations were done in parallel and photograph exposures were equal for sections in human and mice.

The following primary antibodies were used: monoclonal antibodies 6E10 and 4G8 against human Aβ sequence corresponding to residues 1-16 and 17-24, respectively, (Covance Immuno-Technologies, Dedham, MA), Polyclonal A11 specific to AβOs (Biosource, Camarillo, CA), anti-SYP (D4) antibody, monoclonal antibody against β-actin (C4) (Santa Cruz, Santa Cruz, CA), monoclonal anti-drebrin antibody (MBL, Nagoya, Japan), polyclonal anti-PSD-95 (CT) antibody (Invitrogen, Camarillo, CA), and our monoclonal 1A9 and 2C3 antibodies specific to AβOs.

The following secondary antibodies were used (1:1000): Goat anti-rabbit or anti-mouse IgG conjugated with horse-radish peroxidase (HRP) (Invitorogen, Carlsbad, CA), and Goat anti-mouse IgG conjugated with Alex Fluor (AF) 488 or 594 and goat anti-rat IgG conjugated with AF 488 (Molecular Probes, Eugene, OR). Immunopositive signals were visualized using an observation system (Compix Imaging System, Lake Oswego, OR) linked to an Olympus microscope BX50 through a highly sensitive CCD camera or a confocal laser scanning microscope (Carl Zeiss LSM510).

Fluoro-Jade B was purchased from Chemicon (now part of Millipore, Schwalbach, Germany). 7-AAD was purchased from Invitrogen (Carlsbad, CA).

### Histochemistry

For Fluoro-Jade B histochemistry (1:50000), 5-μm-thick paraffin-embedded sections were deparafinized and stained following the manufacturer's instruction (Chemicon, now part of Millipore, Schwalbach, Germany). The Fluoro-Jade B-stained product fluoresces when exited at 488 nm and staining was imaged using a confocal laser scanning microscope (Carl Zeiss LSM510). The same procedure was applied for 30-μm-thick cryostat sections.

### Cell culture and cellular uptake

Human neuroblastoma SH-SY5Y (SY5Y) cells were cultured in Dulbecco's modified Eagle's medium/Ham's F-12 medium supplemented with 10% fetal bovine serum. To investigate the fate of extracellular Aβ, SY5Y cells were exposed to HiLyte Fluor™488-labeled AβMs (10 kDa-filtrate), HiLyte Fluor™488-labeled AβOs (30 kDa-retentate) at 5 μM (AnaSpec, San Jose, CA), or Fluor™488 alone for 10 min, 30 min, and 180 min. In a separate set of experiment, cultures were treated at 37°C for 0, 3, 6, and 24 h with 5 μM Fluor™488-labeled AβOs, and for 24 h with 5 μM Fluor™488-labeled AβMs and synthetic Aβ42-1 (AnaSpec, San Jose, CA). Toxicity was assessed by CytoTox 96 Non-Radioactive Cytotoxicity Assay Kit in accordance with the manufacturer's instructions (Promega, Madison, DI) as described previously [22].

### Protein extraction and immunoblotting

Saline-soluble, saline-insoluble, SDS-soluble fractions, or SDS-insoluble, formic acid (FA)-extractable fractions were prepared from the Tg2576 mouse brains as described previously [32]. Briefly, frozen brain samples were homogenized with a motor-driven Teflon/glass homogenizer (20 strokes) in TBS containing a cocktail of protease inhibitors (150 mg/ml), followed by centrifugation at 100,000 *g *for 1 h. The resultant supernatant (soluble fraction) was subjected to dot blot immunoanalysis or western blotting employing the same antibodies as those used for immunohistochemical staining. The pellet was further extracted with 2% sodium dodecylsulphate (SDS), followed by 70% FA, and the homogenate was ultracentrifuged as described above. The resultant supernatant (insoluble fraction) was also subjected to dot blot immunoanalysis and western blot analysis. For western blotting, aliquots of isolated fractions were separated using NuPAGE 4-12% bis-tris-glycine gels and transblotted to nitrocellulose membrane or Immobilon P (Millipore) for 1 h at 400 mA using 10 mM 3-cyclohexylamino-1-propancsulphonic acid (pH 11) containing 10% methanol. Membranes were blocked for 3 h at room temperature with 5% low-fat milk and 1% BSA in PBST and incubated with either the polyclonal anti-A11 (1:1000) or anti-PSD95 antibody (1:250), and monoclonal anti-drebrin (1:100), anti-SYP (1:2000), and anti-β actin antibodies (1:1000), followed by HRP-labeled goat anti-rabbit or anti-mouse F(ab')_2 _antibody (1:3000; Amersham). Immunoblots were visualized with an ECL kit using LAS3000 mini (Fujitsu, Tokyo, Japan). Densitometric analysis of immunoblot was performed using Multi Gauge v3.0 software (Fuji Film, Tokyo), and bands of interest were normalized to the corresponding actin bands indicated.

### Immunization and behavioral analyses

All animal procedures were performed in accordance with a protocol approved by the Animal Care Committee of the National Institute for Longevity Sciences. Several 3-month-old female nontransgenic (non-Tg) and Tg2576 mice that carry and overexpress the human APP gene with the Swedish double mutation (K670N; M671L) of familial AD were purchased from Taconics (Germantown, NY, USA) and maintained at our animal care facility until 13 months of age. To determine whether immunization prevents the development of Alzheimer-like phenotype, 4-month-old Tg2576 mice were administered 1A9 or 2C3 (0.4 mg/kg/week), or PBS intravenously via the tail vein until 13 months of age. Memory functions were measured at 13 months of age in the following four behavioral paradigms, as described previously [28]: (1) spontaneous alternation in the Y-maze test; (2) novel object recognition test; (3) Morris water maze test; and (4) cued and fear conditioning tests. Mice were sacrificed 3 days after the termination of the behavioral tests for biochemical and histological assessments. Experimental results were analyzed by one-way ANOVA and two-way ANOVA, with Fisher's test for post hoc analysis.

### Spontaneous alternation in Y-maze test

The maze was made of black painted wood; each arm was 40 cm long, 12 cm high, 3 cm wide at the bottom, and 10 cm wide at the top. The arms converged at an equilateral triangular central area that was 4 cm long at its longest axis. Each mouse was placed at the center of the apparatus, the sequence and number of arm entries were recorded for each mouse over an 8 min period. Alternation was defined as successive entry into the three arms on overlapping triplet sets. Alternation behavior (%) was calculated as the ratio of actual alternations to possible alternations (defined as the number of arm entries minus two) multiplied by 100.

### Novel object recognition test

The test procedure consisted of three sessions: habituation, training, and retention. Each mouse was habituated to the box (30 × 30 × 35 cm), with 10 min of exploration in the absence of objects for 3 days (habituation session). During the training session, two objects were placed at the back corner of the box. A mouse was then placed midway at the front of the box and the total time spent exploring the two objects was recorded for 10 min. During the retention session, the mice were placed back in the same box 24 hr after the training session, in which one of the familiar objects used during the training was replaced with a novel object. The animals were then allowed to explore freely for 10 min, and the time spent exploring each object was recorded. Throughout the experiments, the objects used were counterbalanced in terms of their physical complexity and emotional neutrality. A preference index, a ratio of the amount of time spent exploring any one of the two objects (training session) or the novel object (retention session) over the total time spent exploring both objects, was used to measure cognitive function. To eliminate the influence of the last behavioral test, the objects were changed each time.

### Morris water maze test

The Morris water maze test was conducted in a circular pool (120 cm in diameter) filled with water at a temperature of 22 ± 1°C. A hidden platform (one block) (7 cm in diameter) was used. The mice underwent two trials (one block) for 10 consecutive days, during which the platform was left in the same position. The distance taken to locate the escape platform was determined in each trial using the Etho Vision system (Neuroscience Idea Co., Ltd., Osaka, Japan).

### Cued and contextual fear conditioning tests

For measuring basal levels of freezing response (preconditioning phase), mice were individually placed in the conditioning cage (17 × 27 × 12.5 cm) for 1 min, then in the conditioning cage (25 × 31 × 11 cm) for 2 min. For training (conditioning phase), mice were placed in the conditioning cage, then a 15 sec tone (80 dB) was delivered as a conditioned stimulus. During the last 5 sec, an unconditioned stimulus was applied through a shock generator (Neuroscience Idea Co., Ltd.). This procedure was repeated 4 times at 15 sec intervals. Cued and contextual tests were carried out 1 day after the fear conditioning. For the cued test, the freezing response was measured in the neutral cage for 1 min in the presence of a continuous-tone stimulus identical to the conditioned stimulus. For the contextual test, mice were placed in the conditioning cage and the freezing response was measured for 2 min in the absence of the conditioned stimulus.

### Ethics Statement

The research protocol was approved by the local animal esthetics committees at Research Institute, National Center for Geriatrics and Gerontology (Animal Care Committee) prior to initiation of the study. The research project was approved by the local ethics committee of Hirosaki University Graduate School of Medicine, and Research Institute, National Center for Geriatrics and Gerontology prior to initiation of the study.

### Statistical analyses

We used factorial design analysis of variaence (ANOVA) or Mann-Whitney test to analyze data as appropriate. Significant ANOVA values were subsequently subjected to simple main effects analyses or post hoc comparisons of individual means using the Tukey's or Dannett's method as appropriate. We considered *p *values of 0.05 as significant for all studies. Some of the data obtained from animal experiments were analyzed by two-way ANOVA, with Fisher's test for post hoc analysis.

## Competing interests

YO, TY, and MS are the employees in Immunas Pharma Incorporation. TY and MS hold stock options in Immunas Pharma Incorporation. EM, TY, and MS are co-inventors of two filed provisional patent applications titled "Antibody Specific Binding to a Beta Oligomer and The Use" and "Antibodies That Specifically Bind to Aβ Oligomers and Uses Thereof" that cover the antibodies described in this paper, but this does not alter the adherence to all the Molecular Neurodegeneration policies on sharing data and materials. This study has in some parts been funded by a commercial funder, but that does not alter the author's adherence to all the Molecular Neurodegeneration policies on sharing data and materials.

## Authors' contributions

Conceived and designed the experiments: MM EM. Performed the experiments: AT YO TK AM TN HS TU NY EM. Analyzed the data: MS MS KY MM. Contributed reagents/materisals/analysis tools: YO TY MS KA. Wrote the paper: AT EM. All authors read and approved the final manuscript.
